# The impact of inter-organizational alignment (IOA) on implementation outcomes: evaluating unique and shared organizational influences in education sector mental health

**DOI:** 10.1186/s13012-018-0721-1

**Published:** 2018-02-07

**Authors:** Aaron R. Lyon, Kelly Whitaker, Jill Locke, Clayton R. Cook, Kevin M. King, Mylien Duong, Chayna Davis, Mark D. Weist, Mark G. Ehrhart, Gregory A. Aarons

**Affiliations:** 1 University of Washington, 6200 NE 74th Street, Suite 100, Seattle, WA 98115 USA; 2 University of Minnesota, 250 Education Sciences Bldg, 56 East River Road, Minneapolis, MN 55455 USA; 3 University of South Carolina, 1512 Pendleton Street, Columbia, SC 29208 USA; 4 University of Central Florida, 4111 Pictor Lane, Orlando, FL 32816-1390 USA; 5 University of California San Diego, 9500 Gilman Drive (0812), La Jolla, San Diego, CA 92093 USA; 6 Child and Adolescent Services Research Center, San Diego, 92123 CA USA

**Keywords:** Schools, Implementation, Mental health, Inner organizational context, Organizational climate, Inter-organizational alignment, Integrated care

## Abstract

**Background:**

Integrated healthcare delivered by work groups in nontraditional service settings is increasingly common, yet contemporary implementation frameworks typically assume a single organization—or organizational unit—within which system-level processes influence service quality and implementation success. Recent implementation frameworks predict that *inter-organizational alignment* (i.e., similarity in values, characteristics, activities related to implementation across organizations) may facilitate the implementation of evidence-based practices (EBP), but few studies have evaluated this premise. This study’s aims examine the impact of overlapping organizational contexts by evaluating the implementation contexts of externally employed mental health clinicians working in schools—the most common integrated service delivery setting for children and adolescents. Aim 1 is to estimate the effects of unique intra-organizational implementation contexts and combined inter-organizational alignment on implementation outcomes. Aim 2 is to examine the underlying mechanisms through which inter-organizational alignment facilitates or hinders EBP implementation.

**Methods/design:**

This study will conduct sequential, exploratory mixed-methods research to evaluate the intra- and inter-organizational implementation contexts of schools and the external community-based organizations that most often employ school-based mental health clinicians, as they relate to mental health EBP implementation. Aim 1 will involve quantitative surveys with school-based, externally-employed mental health clinicians, their supervisors, and proximal school-employed staff (total *n* = 120 participants) to estimate the effects of each organization’s general and implementation-specific organizational factors (e.g., climate, leadership) on implementation outcomes (fidelity, acceptability, appropriateness) and assess the moderating role of the degree of clinician embeddedness in the school setting. Aim 2 will explore the mechanisms through which inter-organizational alignment influences implementation outcomes by presenting the results of Aim 1 surveys to school-based clinicians (*n* = 30) and conducting semi-structured qualitative interviews. Qualitative data will be evaluated using an integrative inductive and deductive approach.

**Discussion:**

The study aims are expected to identify intra- and inter-organizational constructs that are most instrumental to EBP implementation success in school-based integrated care settings and illuminate mechanisms that may account for the influence of inter-organizational alignment. In addition to improving school-based mental health, these findings will spur future implementation science that considers the relationships across organizations and optimize the capacity of implementation science to guide practice in increasingly complex systems of care.

## Background

Integrated care—defined as care by a team of health professionals, often in non-traditional settings (e.g., primary care, schools) [1]—is becoming increasingly common in adult and youth health and mental health services [2]. In the USA, this growth has been due, in part, to the considerable financial investment of the Affordable Care Act [3]. Similar legislation and policy directives have promoted integrated services internationally [4]. Integrated services have the potential to increase service accessibility and efficiency [5], particularly for the most vulnerable children and adolescents who are disproportionally impacted by fragmented mental health services [6–9]. Nevertheless, the services provided in most integrated care settings could be substantially improved, as they seldom include the delivery of evidence-based practices (EBP) and have failed to consistently show advantages over more traditional services [10–12].

Organizational factors are critical to successful implementation of EBP [13–15], but their unique manifestation in integrated care settings remains unexplored. Research has documented the impact of both general (molar) and implementation-specific organizational factors. Important molar organizational factors include *organizational culture* (i.e., shared values, beliefs, and implicit norms that guide behavior) and *organizational climate* (i.e., employee-shared perceptions of the work environment) [15–17]. Implementation-specific organizational factors include *implementation climate* (i.e., staff’s shared perceptions of the extent to which EBP implementation is expected, supported, and rewarded by their organization), *implementation leadership* (i.e., the attributes and behaviors of leaders that support effective implementation), and *implementation citizenship behavior* (i.e., going beyond the “call of duty” to support implementation) [15, 18–20]. However, most studies are limited to a single organization, and studies of multiple organizations do not examine alignment across organizations. The focus on single organizations does not align with the fundamental nature of integrated care, which often brings together previously disparate service systems with the goal of improving the accessibility and effectiveness of health services [21]. As such, research evaluating the ways integrated care organizations contribute individually and collectively to successful EBP implementation is both overdue and critical to comprehensive understanding of system-level influences.

### Key organizational constructs

The Exploration, Preparation, Implementation, Sustainment (EPIS) framework [2] guides our conceptualization of relevant organizational characteristics and inter-organizational relationships. EPIS identifies critical factors in the inner (i.e., immediate organizational or unit setting where implementation of the innovation takes place) and outer (i.e., more distal settings beyond the immediate setting such as school district, state, federal, etc.) contexts that affect implementation across multiple phases. Inner context factors may be particularly critical to successful implementation and sustainment of EBP [2, 17, 22, 23]. Therefore, this study focuses on inner organizational factors such as general/molar culture and climate and implementation-specific organizational factors (i.e., implementation climate [24], implementation leadership [25], and implementation citizenship behavior). Although the EPIS framework also recognizes the importance of the *inter-organizational context* (connections among organizations or units of the outer and inner settings), few details are specified, reflecting the significant knowledge gap regarding the effects of overlapping organizational contexts on the uptake and delivery of EBP.

A clear understanding of inter-organizational factors that facilitate or hinder implementation is particularly critical in integrated care settings that involve providers who are affiliated with different yet overlapping organizations. EPIS suggests that implementation will be enhanced when overlapping organizations or organizational units exhibit similar values, characteristics, and activities that support implementation, but this prediction has been largely untested. For decades, empirical and practical attention was focused on *coordination* across service sectors, with variable and sometimes negative results [26–28]. Although some studies showed that such coordination improved service access and outcomes [29–31], others found that it negatively impacted quality of care [32]. Qualitative studies support EPIS predictions and suggest that, for organizations to work together successfully, implementation leaders and stakeholders should demonstrate similar core values, shared vision/priorities, and a collective commitment to make a difference [33, 34].

We conceptualize implementation-focused *inter-organizational alignment* (*IOA*) as the extent to which these commonalities manifest across organizations in key implementation constructs and across system or organizational levels. For example, regarding implementation climate, alignment in integrated care settings may manifest as shared expectations between organizations surrounding the delivery of high-quality services. *Intra*-organization consistency in the messages communicated to employees results in decreased employee confusion and facilitates employee internalization of organizational objectives [35–39]. It follows that *inter*-organizational alignment in implementation climate would have similar effects for employees who work in integrated care settings. Importantly, although a number of qualitative studies have supported the importance of alignment in implementation climate for EBP adoption [34, 40, 41], its influence—and the relative influence of different organizational factors—have yet to be examined quantitatively.

As a second example, strong organizational leadership frequently emerges in qualitative studies as a critical component of successful inter-organizational collaborations [40–42]. When the leaders of organizations that collaborate to provide integrated care both support and engage in behaviors that facilitate implementation, this is likely to create a synergistic climate of openness, support, and accountability. However, to our knowledge, there are no quantitative studies regarding the combined impact of implementation leadership from multiple organizations. This study will explore the impact of alignment of these and other organizational constructs on implementation.

### Inter-organizational alignment in school-based mental health

Schools provide convenient access to services and reduce barriers to treatment typically seen in traditional outpatient settings. This is particularly true for vulnerable ethnic and economic minority groups [43–46]. In part for this reason, school-based mental health (SBMH) programs have progressively grown in the USA and abroad [47–49], to the point where studies consistently indicate that 50–80% of all youth mental health services are provided in schools [50–52]. Despite the practicality and pervasiveness of SBMH, implementation of EBP in schools is highly variable, dramatically reducing the public health impact of these services [53–55]. Limited research has evaluated intra-organizational influences on the implementation of EBP in SBMH [56]. Furthermore, as with most other integrated care settings, very little work has evaluated the influence of inter-organizational factors on the delivery of evidence-based SBMH services.

**Fig. 1:**
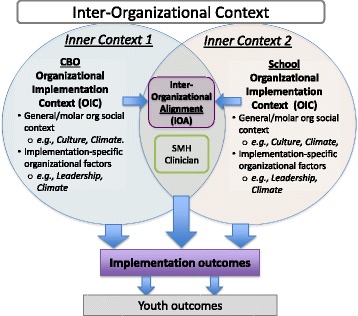
Inter-organizational context and inter-organizational alignment in school mental health

Most frequently, SBMH services are provided by clinicians who are trained and employed by community-based organizations (CBOs) that exist outside of the education system [57]. This can lead to substantial administrative and contextual differences between schools and mental health systems (e.g., training, funding, etc.) that may impact the provision of services [58]. For example, in looking at mental health staff employed by schools (e.g., school psychologists, counselors) as compared to staff from CBOs working in schools (e.g., clinical social workers, psychologists), there are significant differences in areas such as their ability to obtain consent for services, providing effective interventions, team functioning, and confidentiality standards [59]. This dual administrative relationship is one that makes SBMH particularly conducive to studying the impact of IOA and generalizable to other integrated service settings [60]. Figure 1 displays our conceptualization of the inter-organizational context for SBMH. A series of studies has provided initial support for the influences of *both* the school and CBO organizational contexts. For instance, CBO factors significantly impact participation in post-training consultation and, ultimately, implementation success, even when accounting for clinician-level variables (e.g., EBP knowledge, attitudes) [61]. There also is evidence to suggest that the organizational context of schools predicts the use of EBP in public schools, with subsequent qualitative findings suggesting that implementation outcomes may be a function of both the CBO and school organizational contexts [62]. As a whole, the existing research indicates that each context plays an important role in successful implementation in schools, but it is unknown whether overlapping contexts have an interactive effect on implementation outcomes [11, 63].

#### Clinician school embeddedness

The relative influence of school and CBO contexts also is likely to be affected by the extent to which SBMH clinicians are strongly connected to and collaborate with school personnel, which we term *organizational embeddedness.* Some clinicians are intimately involved in school functions, while others see students in isolation from other aspects of the school [64, 65]. Further, social relationships can motivate colleagues to support new initiatives and demonstrate commitment even when implementation is difficult [40, 66]. Well-embedded clinicians are more likely to be connected with opinion leaders or other implementation facilitators. Because it reflects a more extensive social connection to the school and school-based personnel, high clinician embeddedness may strengthen the impact of the school context on implementation outcomes. This embeddedness is influenced by school level factors, such as principal support for mental health services, and district level factors such as memoranda of agreement between schools and community agencies that sanction CBO clinician involvement in teams and other functions [67]. To date, no research has examined the moderating role of embeddedness in an inter-organizational setting.

### Mechanisms of influence

Improving the efficiency, speed, and sustainment of EBP implementation requires research on the mechanisms through which multiple organizations impact implementation outcomes, yet no empirical evidence on these mechanisms exists to date [2, 14, 68, 69]. However, candidate mechanisms for IOA at the organizational and individual levels can be identified to facilitate research, tailor implementations to context, or identify core components to increase the precision of implementation strategies [70]. When considering the partnership between two organizations, mechanisms may include group-level processes drawn from established organizational frameworks, such as *inter-organizational fragmentation* or *cohesion* [71, 72]. At the individual level, the recently articulated Research Domain Criteria (RDoC) [73, 74] may provide a template for identifying potential mechanisms that explain how inter-organizational processes influence the implementation behavior of individual employees. Although the RDoC were developed primarily to elucidate the processes through which psychopathology is developed or maintained, they provide a more general way of organizing factors that may explain the impact of environmental influences on individual outcomes. These include variables such as the impact of *positive valence systems* (e.g., reward learning, habit formation) that increase the use of new skills in response to consistent organizational supports (e.g., supportive leadership), *negative valence systems* (e.g., confusion and dread) that may result from misaligned culture or climate, or *social processes* (e.g., perception and understanding of others) that explain how consistent organizational norms or employee behaviors influence clinician engagement with EBP in well-aligned settings. The second aim of this study (below) will fill a substantial knowledge gap by exploring the underlying mechanisms through which inter-organizational factors facilitate or hinder implementation.

### Objectives and aims

The long-term objective of this research is to facilitate a more detailed understanding of how inter-organizational linkages operate to facilitate—or impede—implementation of EBP in integrated settings. To achieve this objective, this study has two aims.

Aim 1

Conduct quantitative surveys with school-based, externally employed mental health clinicians, their supervisors, and proximal school staff (i.e., those whose roles give them familiarity with school-based mental health services) to estimate the effects of intra-organizational variables and inter-organizational alignment on implementation outcomes (fidelity, acceptability, appropriateness) and assess the moderating role of clinician embeddedness (i.e., degree to which the person is visible and interacts with others in the setting). Aim 1 will yield quantitative information about inter-organizational alignment to be further explored in Aim 2.

Aim 2

Explore the mechanisms through which inter-organizational alignment influences implementation outcomes by presenting the quantitative findings from Aim 1 to school-based clinicians and eliciting qualitative feedback regarding hypothesized mechanisms that may drive quantitative associations between organizational factors and implementation outcomes. Aim 2 will yield novel information about the pathways through which organizational processes in integrated settings influence the implementation outcomes assessed, contributing to the nascent literature on implementation mechanisms more generally [75] and advancing future research endeavors to tailor implementation strategies to a given context.

## Methods/design

An observational design will be used to examine intra- and inter-organizational factors that influence successful implementation of EBP in SBMH, followed by a mixed-methods approach to elucidate mechanisms of influence. This will include (1) large-scale administration of a web-based survey to school and CBO personnel to estimate the effects of school and CBO organizational contexts, as well as their alignment, and (2) a mixed methods approach [33, 76] in which qualitative data are collected about quantitative data to identify the mechanisms linking inter-organizational characteristics and alignment to implementation outcomes.

### Participants

Participants in both aims will be SBMH providers and proximally related professionals employed by CBOs and elementary, middle, and high schools located in two large urban school districts in the Midwest and Pacific Northwest. Proximal personnel within CBOs include SBMH clinicians and supervisors, whereas those in schools may include professionals such as school nurses, counselors, social workers, or administrators. Sites were selected for having longstanding SBMH programs (which are expected to yield a range of clinician embeddedness), administrative relationships with the schools reflecting the most common arrangements nationally (i.e., CBOs providing school-based services via a county contract), and substantial ethnic and economic diversity of the district student bodies (60%+ nonwhite; 45–65% low income). Based on our team’s prior research and the demographic makeup of the participating organizations, it is expected that school and CBO personnel will be approximately 80–85% female and 75% Caucasian, 10% African American, 8% Asian, and 10% Latino/a. The total sample will be 120 participants, including 30–35 clinicians and 90 school personnel.

### Aim 1 design: quantitative evaluation of intra- and inter-organizational contexts

#### Procedures

To address Aim 1, measures will be collected via web-based survey. To ensure high response rates, backup data collection procedures include in-person surveys, mailed surveys, and telephone follow-up. All CBO-employed SBMH clinicians will be recruited from each site. To identify school staff with proximal roles to social, emotional, and behavioral programming in schools, survey administration will be sequential over two phases with school-based clinicians completing the first phase of data collection. During their survey completion, clinicians will identify staff from their schools who have roles that include supporting student social, emotional, and behavioral health (e.g., school nurse, school psychologist, school counselor, teacher lead for student behavioral supports). Investigators will then contact each individual by email and/or telephone to introduce the study and answer any questions. Consent will be obtained as part of the web survey.

#### Measures

Consistent with prior organizational research [77], we will obtain at least three employee ratings for each of the three general/molar and three implementation-specific organizational-level constructs from personnel most proximal to the delivery of school-based mental health services. Because they are embedded in both contexts, SBMH clinicians will complete measures for both CBOs and schools. To avoid sequencing biases, clinicians will be randomized as to whether they first complete questions about their CBO or their school. Scores will be aggregated across reporters to yield scores for each organization on organizational measures. As detailed below, the organizational implementation context of CBOs will be measured using well-established tools of the organizational social context and implementation-specific organizational factors. The school organizational context will be evaluated using versions of those tools that have previously been adapted for use in schools [56, 78]. Individual-level covariates (i.e., attitudes, citizenship) will also be collected using school-adapted instruments. Implementation outcomes will be gathered from SBMH clinicians and supervisors.

##### Demographics

A demographic survey for all participants (school and CBO) will include age, gender identity, race/ethnicity, education, years of training/terminal degree completion, primary discipline, experience, and work setting/activities performed.

##### Organizational social context (OSC)

The OSC is a measure of general/molar organizational culture and climate [15]. In the current study, only three subscales: Proficiency, Stress and Functionality will be administered; subscale *α* = 0.78–0.94.

##### Implementation Leadership Scale (ILS)

The ILS, a measure of implementation leadership [25, 79], has 12 items loading onto four subscales: Proactive Leadership, Knowledgeable Leadership, Supportive Leadership, and Perseverant Leadership; subscale *α* = 0.95–0.98.

##### Implementation Climate Scale (ICS)

The ICS, a measure of implementation climate [24, 80], includes 18 items loading onto a total score and six subscales: Focus on EBP, Educational Support for EBP, Recognition for EBP, Rewards for EBP, Selection for EBP, and Selection for Openness; subscale *α* = 0.81–0.91.

##### Implementation Citizenship Behavior Scale (ICBS)

The ICBS is a measure of an individual’s implementation citizenship behavior (i.e., the extent to which employees go “above and beyond the call of duty” for EBP implementation) [81] and consists of six items loading onto two subscales: Helping Others and Keeping Informed; subscale *α =* .94–.95 [56].

##### Attitudes toward evidence-based practices scale (EBPAS)

Clinician attitudes toward evidence-based practice will be measured using a version of the EBPAS [82] that was adapted for use with education sector service providers and consists of 16 items that load onto four subscales: Requirements, Appeal, Openness, and Divergence; subscale *α =* .59–.90.

##### Expanded school mental health collaboration instrument (ESCI)

The ESCI [64] will be used to evaluate both clinician embeddedness and mental health service quality. The Outreach and Approach by Mental Health Professionals subscale includes 13 items and assesses the level of embeddedness of community-mental health clinicians in schools. Improvements in school mental health delivery will be assessed with three additional subscales from the ESCI: Support for Teachers and Students (how students and teachers are supported through SBMH programming, 8 items); Increased Mental Health Programming, (improvements in services at school, 5 items); and Improved Access for Students and Families (3 items); subscale *α =* .84–.94.

##### Fidelity/integrity

Consistent with prior organizational research [81], implementation integrity will be evaluated via a modified set of four items completed independently by clinicians and supervisors. Integrity items address practices used with clients, competence, and adherence. Internal consistency for these ratings has been found to be high (*α* = .97) [81]. As final component of this measure, respondents also will provide information about the specific EBP that they have implemented in their work setting.

##### Acceptability and appropriateness

Acceptability and appropriateness of new EBP will be assessed using the four-item Acceptability of Intervention Measure (AIM) and Intervention Appropriateness Measure (IAM) [83], tailored to ask generally about evidence-based practices. The measures have been found to have good inter-item reliability (0.85 for acceptability, 0.91 for appropriateness) and test-retest reliability (0.83 for acceptability, 0.87 for appropriateness).

#### Analyses

Basic data screening procedures will be conducted to screen for errors and explore normality, linearity, form, and outliers. Data will be transformed as appropriate. Statistical analyses will be conducted using commonly available software packages (SPSS [84], Stata [85], Multilevel/Hierarchical Level Modeling [MLM/HLM] [86], and R [87]).

##### Inter-organizational alignment

IOA is the degree to which schools and CBOs exhibit similarity on organizational variables linked to implementation (e.g., general/molar organizational culture; implementation leadership). To describe variability in alignment, we will first use the intra-class correlation coefficient, which estimates variability in each variable that is common across schools and CBOs. Prior research has suggested that difference scores are not ideal as a measure of agreement because they provide no information about whether the effect of a difference depends on the level of either variable [88, 89]. Thus, IOA will be analyzed using polynomial regression with response surface analysis [90, 91] described below.

##### Modeling approach

A multilevel modeling (MLM) framework will allow us to account for the fact that approximately 35 SBMH clinicians in approximately 25–30 schools will be nested within 10–12 CBOs. Because this design will capture nearly all of the school-serving CBOs in two regions, analyses will provide near population level estimates of effects. Thus, analyses will focus on obtaining effect size estimates rather than on traditional hypothesis testing; these estimates will be used to inform the qualitative aims. We will use the estimated effects from these models to guide the development and design of qualitative interview guides addressing organizational factors found to most strongly predict implementation outcomes. We will first estimate the effects of each measure of the organizational implementation context (e.g., general/molar organizational climate, implementation climate) on each implementation outcome, using CBO and school organizational variables as unique predictors of clinician level implementation outcomes. We also will include individual variables (e.g., EBP attitudes) in the multilevel models as level 1 (i.e., clinician level) covariates, testing whether their inclusion improves model fit via likelihood-ratio testing. Next, we will examine the effects of IOA in two ways. First, for each variable, we will model an interaction between CBO and school context; the size of the interaction provides an estimate of the *interdependent* effects of the school and the CBO organizational variables. We also will examine the effects of alignment using the polynomial regression with response surface analysis approach [92], which examines nonlinearity of alignment effects (e.g., effects of alignment when organizational variables are rated similarly in both organizations) and allows a visual examination of the effects of alignment. Finally, we will estimate the effects of our organizational variables on embeddedness for each organization (school or community-based organization) independently, estimate the effects of inter-organizational alignment on embeddedness, and examine the moderating effects of embeddedness on the separate influences of the school and CBO contexts. Overall, this analytic approach will enable us to obtain effect size estimates of the inter-organizational factors that impact clinician implementation outcomes, which will be used as the foundation for the qualitative phase of the project.

##### Power

Our study is largely focused on estimating effects of an entire population rather than computing statistical significance or generalizing to a larger population and therefore potentially less susceptible to issues associated with calculating effect sizes in small samples [93, 94]. Nevertheless, we calculated power to detect effects in MLM by computing a “corrected sample size,” which adjusts the sample size by the correlation due to nesting [68, 95]. Assuming an ICC of .10 [96], our effective sample size to detect effects on clinician-level implementation outcomes with 35 clinicians would be *n* = 29. Thus, the proposed study has the sensitivity at power (1-*β*) = .80 (*α* = .05) to detect effects as small as *f*^2^ = .37.

### Aim 2: identification of preliminary implementation mechanisms

#### Procedures

Qualitative and mixed-methods inquiry is critical to mechanistic research [97]. To address Aim 2, visual summaries of Aim 1 quantitative, organizational data will be presented to all SBMH clinician participants to evaluate the ways in which alignment/misalignment of the CBO and school contexts influences their implementation of new practices. Because the objective is to describe the mechanisms through which IOA exerts its influence on SBMH clinicians, the perspectives of participants who directly experience both organizations (i.e., SBMH clinicians) are most critical. Individual semi-structured phone interviews, lasting approximately 45–60 min, will be conducted at a convenient time for clinicians and audio-recorded. Recordings will be transcribed prior to coding. The mixed methods design will be *sequential in structure* (quantitative data in Aim 1 collected prior to qualitative data in Aim 2); the functions are *explanation* and *expansion* (we will use qualitative data to provide depth and breadth of understanding to explain the mechanisms that explain the influence of IOA on implementation, i.e., QUAN➔QUAL), and the process is *connecting* (the qualitative dataset will build on the quantitative dataset) [35, 76, 98, 99]. Qualitative data in Aim 2 will explore the nuances of inter-organizational processes to understand mechanisms that affect implementation.

#### Qualitative interview protocol

We will develop a systematic, comprehensive semi-structured interview guide that examines underlying mechanisms through which inter-organizational processes facilitate or hinder EBP implementation. Feedback reports will be presented to participants for their respective school-CBO dyad and will include all of the organizational constructs measured in Aim 1 (i.e., OSC, ILS, ICS). Using these constructs as an overarching framework, we will generate questions that explore the inter-organizational implementation context as perceived by CBO clinicians and supervisors. Specifically, a series of questions will (1) request examples of how alignment/misalignment on each construct manifests in their work settings and (2) explore the mechanisms/processes through which degree of alignment/misalignment affects their ability to implement EBP. Questions will be carefully constructed to elicit clear information without assigning valence to performance. We also will ask questions about clinician embeddedness to gain a more nuanced understanding of the implications of clinician connections to and collaborations with the schools in which they work. Synthesis of this information will point to potential mechanisms through which IOA impacts successful implementation.

#### Analyses

All interviews will be transcribed and imported into the software package NVivo [100]. Data will be coded using an integrated deductive and inductive approach as certain codes will be conceptualized during the interview guide development (i.e., deductive approach), and other codes will be developed through a close reading of an initial subset of transcripts (i.e., inductive approach) [101]. These themes will provide a way of identifying and understanding potential mechanisms through which IOA impacts implementation [102, 103]. Mechanisms can be both facilitative (i.e., those that result in better implementation) and inhibitory (i.e., those that impede implementation) and will be compared to a variety of individual and organizational mechanistic frameworks [71, 72, 74]. After a stable set of codes is developed, a consensus process will be used in which all reviewers independently code all of the transcripts and meet to compare their coding to arrive at consensus judgments through open dialog [104–106]. Consensus coding is designed to capture data complexity, avoid errors, reduce groupthink, and circumvent some researcher biases. Mechanisms identified via qualitative analyses will be summarized separately for each organizational construct to determine the specific factors that facilitate or inhibit implementation based on alignment relationships.

#### Sample size/power

Based on our previous work [107], it is anticipated that nearly all clinician participants from Aim 1 also will participate in qualitative interviews, resulting in a sample size of approximately *n* = 30. Based on our previous research focused on organizational issues in schools [66, 108], we anticipate that this total will be adequate to achieve data saturation and capture the constructs of interest [109, 110].

## Discussion

### Innovation

This study will address significant gaps in implementation science knowledge and is innovative by simultaneously examining the organizational contexts of multiple settings (schools and CBOs providing mental health services in schools), linking inter-organizational alignment to implementation, assessing the role of clinician embeddedness, and evaluating the mechanisms through which these processes impact clinician implementation outcomes. Previous implementation research in SBMH has been almost entirely qualitative and has focused on how the organizational factors of *either* the school [111, 112] *or* the CBO [113, 114] individually affect implementation. Notwithstanding the contributions of this research, conducting research in silos does not capture the dynamic and interactive nature of organizations and the influence they have on the delivery of high-quality integrated care. No research has simultaneously examined the unique organizational contexts of both schools and CBOs involved in the implementation of mental health EBP. This neglects the dual administrative relationship that typifies SBMH, where services are most commonly provided by clinicians who are trained and employed outside of the education system [57]. Examining the impact of these organizations simultaneously not only has potential to improve implementation science in SBMH but may be generalizable to other integrated care settings.

Further, contemporary implementation frameworks typically assume a single organization within which system-level processes influence service quality and implementation success. This perspective does not represent the emerging realities of modern mental health care [3, 4] and inhibits what can be learned about organizational processes in implementation. This study is the first to examine the specific impact of IOA in an integrated care setting. Findings have the potential to spur future implementation science that considers the complex relationships across organizations, inform efforts to design and tailor implementation enhancement interventions that attend explicitly to the inter-organizational context, and optimize the capacity of implementation science to guide practice in increasingly complicated systems of care.

Despite growing emphasis on mechanisms of action in both intervention science [73, 74, 97] and implementation science [75], no research has evaluated the mechanisms through which overlapping organizational contexts impact implementation. This study will use mixed methods to advance the field’s nascent understanding of mechanisms by exploring the pathways through which IOA influences implementation outcomes. Explicitly evaluating these mechanisms represents a significant step forward for implementation research that seeks to develop more precise strategies that target the processes through which organizational factors impact implementation outcomes.

Finally, because the level of SBMH clinician embeddedness in schools reflects their communications, collaborations, and relationships with school staff [115], this study explores the moderating role of SBMH clinician embeddedness on implementation. In SBMH, there is considerable variability in the extent to which clinicians are connected to and collaborate with school personnel [64, 65]. This provides a unique opportunity to examine how organizational influences in general—and IOA in particular—might vary by level of embeddedness.

### Limitations

First, the current project is designed to estimate the effects of inter- and intra-organizational variables on implementation outcomes but is not powered for traditional significance testing within a multilevel framework. Recent work shows that studies with less than optimal statistical power can produce important scientific findings and result in more scientific value per dollar spent than larger sample sizes [116]. Further, null hypothesis testing yields less information about the potential reproducibility of study findings than parameter estimates and accompanying confidence intervals [117, 118]. Consequently, we have framed our research questions and design—for both main and moderating effects—in terms of the magnitude, direction, and precision of effect estimates in order to determine the plausibility of effects. There are approximately 13 CBOs operating in the two participating school districts in which we will recruit participants. We intend to include 90–100% of them in the current study, providing an estimate of effects that approximates the population parameters for these regions.

Second, this study is cross-sectional. We considered a longitudinal design that would evaluate changes in organizational constructs (and their alignment) at multiple time points but determined that the limited literature on IOA, objective of estimating effects, and resource limitations were most conducive to a cross-sectional design. Third, although objective indicators of some implementation outcomes (e.g., integrity) may be ideal, objective outcomes that would be common across agencies, schools, and EBP are not available. Instead, subjective and generalizable individual- and team-level EBP implementation outcome measures were drawn from prior research conducted by members of the study team [24, 79]. These measures allow for responses from multiple informants, which have previously been found to overcome some self-report/common method biases [119, 120].

### Conclusion and impact

Assessment of organizational processes in implementation has fallen behind the realities of contemporary integrated healthcare, which often spans multiple providers, settings, and organizations. Given the well-established importance of organizational processes for the success of implementation initiatives, simultaneous attention to multiple, overlapping settings is likely to become increasingly relevant. The sequential findings yielded from the current aims are intended to inform research both within and beyond the education sector as the field of implementation moves increasingly toward mechanistic inquiry as a pathway to identifying the most parsimonious and effective implementation strategies.

## Abbreviations

AIM: Acceptability of Intervention Measure
CBO: Community-based organization
EBP: Evidence-based practice
EBPAS: Evidence-Based Practice Attitudes Scale
EPIS: Exploration, Preparation, Implementation, Sustainment
HLM: Hierarchical linear modeling
IAM: Intervention Appropriateness Measure
ICBS: Implementation Citizenship Behavior Scale
ICS: Implementation Climate Scale
ILS: Implementation Leadership Scale
IOA: Inter-organizational alignment
MLM: Multilevel modeling
OIC: Organizational implementation context
SBMH: School-based mental health
